# Polymorphisms in *FSHR* modulating susceptibility to polycystic ovary syndrome: an updated meta-analysis

**DOI:** 10.1186/s13048-023-01238-7

**Published:** 2023-09-01

**Authors:** Mandeep Kaur, Sukhjashanpreet Singh, Anupam Kaur

**Affiliations:** Department of Human Genetics, Guru Nanak Dev University, Amritsar, Punjab 143005 India

**Keywords:** PCOS, *FSHR*, Meta-analysis, Genetic models, Polymorphisms

## Abstract

**Background:**

Two polymorphisms, rs6165 and rs6166 located in the intracellular domain of FSHR has been reported to affect folliculogenesis, steroidogenesis and oocyte maturation. Several studies have highlighted the role of *FSHR* polymorphisms in PCOS but the findings are conflicting. A meta-analysis was carried out to decipher the emerging perspectives.

**Methodology:**

A comprehensive literature search was made using PubMed, PCOSkb, and Google Scholar. New Ottawa Scale has been utilized to evaluate the quality of each article. To evaluate the strength of association under different genetic models of rs6165 and rs6166 polymorphisms, odds ratio with a 95% confidence interval (CI) was calculated.

**Results:**

A total of 20 articles were selected for the present study. In pooled analysis and after the stratification by ethnicity, polymorphism rs6165 remains unrelated to the onset of PCOS. Besides, rs6166 exhibits significant protection in the Indian population under recessive, additive, and allele models (OR = 0.7, CI: 0.54–0.9, *p* = 0.006, OR = 0.65, CI: 0.48–0.89, *p* = 0.006, OR = 0.82, CI: 0.7–0.95, *p* = 0.01, respectively) and low to moderate risk in the Caucasian population under allele model (OR = 1.17, CI: 1.04–1.32, *p* = 0.01).

**Conclusion:**

This meta-analysis suggests that GG genotype of rs6166 provides protection against PCOS, in a population-specific manner.

## Introduction

Polycystic ovary syndrome (PCOS) is a complex endocrine disorder affecting females of reproductive age and is the foremost cause of anovulatory infertility [1, 2]. The worldwide prevalence of PCOS is between 6 and 26% [3]. Chronic anovulation, hyperandrogenism, and menstrual irregularities are the characteristic features of PCOS [4], which are additionally accompanied by obesity, insulin resistance and high LH levels [5, 6]. The diagnosis of PCOS is based on Rotterdam criteria 2003 which states that 2 out 3 features: a) Oligo/anovulation, b) clinical or biochemical sign of hyperandrogenism, and c) presence of polycystic ovaries on ultrasonography should be present [7]. It is becoming evident that PCOS can affect a woman anytime. It may start while she is still in the womb and show clinical signs in adolescence which continue throughout her reproductive years. Moreover, even after menopause, PCOS women are more likely to develop metabolic diseases like diabetes, hypertension, and cardiovascular disease [8].

The precise etiology of PCOS is yet unknown, but it was suggested that the interplay of genetic and environmental factors is responsible for this condition [9]. In PCOS, the levels of gonadotropins such as follicle-stimulating hormone (FSH), luteinizing hormone (LH) and prolactin are abnormal. Due to persistently high-frequency GnRH stimulation, women with hyperandrogenic PCOS exhibit an increased LH pulse frequency and low FSH [10]. Lower FSH levels result in follicular development arrest, contributing to ovulatory dysfunction in PCOS. These alterations in gonadotropin secretion (LH and FSH) in PCOS may also depend on genetic variants of gonadotropic-related genes such as FSHR (FSH receptor) and LHCGR (LH/choriogonadotropin receptor) and are supported by various studies [11].

The FSHR is located on chromosome 2 at position p21–p16 and has 10 exons and 9 introns. The extracellular domain of the receptor is encoded by the first 9 exons. In contrast, the C-terminal end of the extracellular domain, the whole transmembrane domain, and the intracellular domain of the FSHR are all encoded by exon 10. In females, FSHR is expressed in granulosa cells and regulates the development of graffian follicles, granulosa cell proliferation and estrogen synthesis [12]. When FSH binds to its receptor, FSHR, it activates a number of intracellular signalling pathways, and for signal transduction exon 10 is crucial [13, 14]. Mutations in *FSHR* specifically in exon 10 can lead to the arrest of follicle development at several phases of growth and has several effects on phenotype such as variable development of secondary sex characteristics, primary amenorrhea, hypoplastic ovary and high serum levels of FSH [15, 16]. Ser680Asn (rs6166) and Ala307Thr (rs6165) are the two polymorphisms located in the exon 10 of *FSHR* and are well known to affect the efficacy of FSHR receptor towards its ligand (FSH), increase FSH levels in a compensatory manner. This increases FSHR resistance leading to reduce estrogen and inhibin B that establish the inhibitory feedback loop in the pituitary gland, resulting in hyperandrogenism which may arrest follicle development [17]. According to different studies, these polymorphisms may affect the menstrual cycle, ovarian hyperstimulation and PCOS development [18, 19]. Several studies have been carried out across the globe to e the genetic association of these SNPs but the results were conflicting. In order to resolve differences in genetic association research, meta-analysis has been a widely known method. It specifically incorporates findings from various studies on the same subject, improving statistical strength and accuracy in effect estimation [20]. Although, meta-analysis has already been done earlier on both variants [21–23], however, there are some additional publications on *FSHR* polymorphisms [24, 25]. Furthermore, a recent meta-analysis by [23] includes only Asian studies. Hence, in order to ascertain the relationship between these polymorphisms and PCOS susceptibility in the global population, we further conducted a thorough and updated meta-analysis.

## Material and methodology

### Search strategy

Comprehensive computer-based literature searches on Google Scholar, PubMed, and PCOSkb were used to find each study that has reported the genetic association of Polycystic Ovary Syndrome and *FSHR* polymorphisms (rs6165 and rs6166) without any language barrier (up to March 2023). The following set of MeSH keywords and terms were used: Polycystic Ovary Syndrome or PCOS or Stein Leventhal Syndrome; FSHR or Follicle Stimulating Hormone Receptor or FSH; rs6165 or rs6166 or Ala307Thr or Ser680Asn; gene or allele or genotype or mutation or variant or variation or polymorphism or Genetic variant or Genetic variation. Moreover, manual screening was done on the reference lists of research articles and earlier meta-analyses.

### Inclusion/ exclusion criteria

In this meta-analysis, studies fulfilling the following criteria were included: (a) a case–control design (b) evaluation of the association of rs6165 and rs6166 with PCOS (c) genotype frequency of controls in the Hardy Weinberg equilibrium (HWE) (d) provides genotypic data for both cases and control group (e) studies on human blood samples (f) published in the English language. Exclusion criteria were as follows: (a) not the case–control design, (b) Controls genotype frequency deviated from HWE (c) The design is based on family or sibling pairs (d) Animal studies.

### Data search and quality assessment

Data were extracted from selected publications based on inclusion criteria. From each study, the following information was gathered: first Author name, year of publication, country of origin, diagnostic criteria of PCOS, method of genotyping, the total number of cases and controls and evidence of HWE in controls.

To check the quality of each publication included in the present meta-analysis New Castle Ottawa scale (NOS) [26] was used. NOS is based on a star scoring system and is categorized into three parts: a) Selection b) Comparability c) Outcome. For the non-randomized meta-analysis, each publication can be given a total of 9 stars, with 0 to 3 stars, 4 to 6 stars, and 7 to 9 stars representing low, moderate, and high quality, respectively. For the current meta-analysis, publications of moderate and high quality were chosen, while publications of low quality were excluded. Finally, PRISMA 2020 (Preferred Reporting Items for Systematic Reviews and Meta-Analysis) checklist and flow diagram were used for this meta-analysis.

### Statistical analysis

The power of the study was calculated using a Cats-power calculator which rendered the power to be > 95%. A goodness of fit Chi-square calculation was used to determine any deviations from HWE. Pooled odds ratios (OR) and 95% confidence intervals (CIs) were used to evaluate the strength of the association for the meta-analysis. The association was determined using the following four genetic models: dominant model (GG + AG vs. AA), recessive model (GG vs. AG + AA), additive model (GG vs. AA), and allele model (G vs. A). To assess heterogeneity, the I^2^ statistic was used and a random effect model (REM) was chosen when I^2^ was greater than 50%, indicating that heterogeneity is present, while a fixed effect model (FEM) was chosen when I^2^ was less than 50%, indicating that heterogeneity was absent. To assess publication bias, Beggs’s funnel plot was used. All data were analysed using Review Manager 5.4.1. Bonferroni correction was applied to *p*-value in order to reduce the type 1 error.

## Results

### Studies included in the meta-analysis

**Fig. 1:**
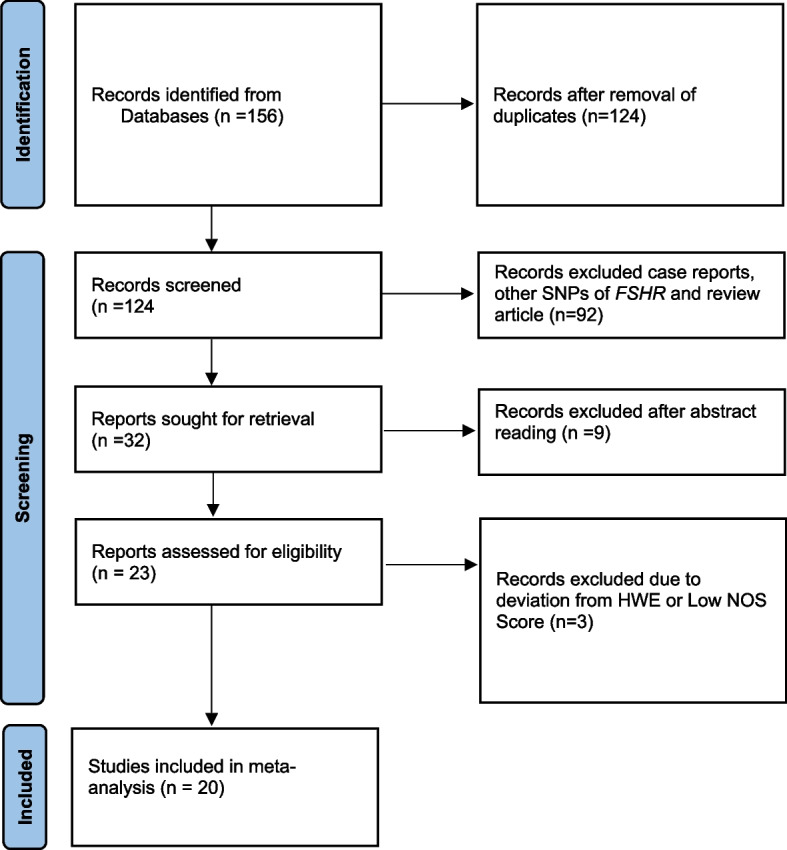
Flow diagram of the selection process for meta-analysis (according to PRISMA guidelines)

Figure 1 displays the flowchart for the search process and search results. A total of 156 possible studies were gathered through the use of database search and manual search. Titles and abstracts were carefully examined and 124 papers were eliminated, because they were duplicates, review articles, or case reports. Following full-text analysis, 10 were excluded since they were not case–control studies designed or have enough information for meta-analysis. In addition, two studies that deviated from Hardy Weinberg equilibrium were excluded, and one study was also taken out since it had a low NOS score. There were no additional relevant studies found despite our search of recent reviews and meta-analyses. Eventually, 20 case–control studies were selected for meta-analysis, and of these 20 studies, 16 were on Asians and 4 studies were on Caucasians. Table 1 enlists the distinguishing characteristics of all selected studies. Genotype frequency and HWE *p*-value of included studies were tabulated in Table 2.

**Table 1:** List of included studies in the present meta-analysis

Sr. No	Study	Year	Country	PCOS diagnostic criteria	Variants Studied	Genotyping method	Sample size (Cases/Controls)
1)	Conway et al. [27]	1999	UK	PCO + OA + MD	rs6165, rs6166	PCR-SSCP	93/51
2)	Tong et al. [28]	2001	China	HA + PCO + MD	rs6165	PCR–RFLP	124/236
3)	Sudo et al. [29]	2002	Japan	Rotterdam Criteria	rs6165, rs6166	PCR–RFLP	18/168
4)	Unsal et al. [30]	2009	Turkish	Rotterdam Criteria	rs6165, rs6166	PCR–RFLP	44/50
5)	Valkenburg et al. [17]	2009	Netherlands	Rotterdam Criteria	rs6166	PCR-SSP	495/2912
6)	Du et al. [31]	2010	China	Rotterdam Criteria	rs6165, rs6166	PCR-SSP	55/92
7)	Gu et al. [16]	2010	Korea	Rotterdam Criteria	rs6165, rs6166	PCR–RFLP	235/128
8)	Mohiyiddeen et al. [32]	2012	UK	Rotterdam Criteria	rs6166	Taq man assay	58/83
9)	Fu et al. [33]	2013	China	Rotterdam Criteria	rs6165, rs6166	Sequencing	384/768
10)	Kambalachenu et al. [34]	2013	India	Rotterdam Criteria	rs6166	PCR–RFLP	97/101
11)	Liaqat et al. [35]	2013	Pakistan	Rotterdam Criteria	rs6165, rs6166	PCR-SSP	96/96
12)	Singhasena et al. [36]	2014	Thailand	Rotterdam Criteria	rs6165, rs6166	PCR–RFLP	133/132
13)	Wu et al. [37]	2014	China	Rotterdam Criteria	rs6165, rs6166	PCR–RFLP	215/205
14)	Almawi et al. [38]	2015	Bahrain	Rotterdam Criteria	rs6166	Real-time PCR	203/211
15)	Thathapudi et al. [39]	2016	India	AES	rs6166	PCR–RFLP	204/204
16)	Kim et al. [4]	2017	Japan	Rotterdam Criteria	rs6165, rs6166	Sequencing	377/388
17)	Branavan et al. [40]	2018	Sri Lanka	Rotterdam Criteria	rs6165, rs6166	Real-time PCR	55/110
18)	Wan et al. [23]	2021	China	Rotterdam Criteria	rs6165, rs6166	Sanger sequencing	400/480
19)	Kaur et al. [24]	2023	India	Rotterdam Criteria	rs6165, rs6166	PCR–RFLP	421/322
20)	Vieira et al. [25]	2023	Portugal	Rotterdam Criteria	rs6166	PCR–RFLP	88/80

*PCO* Polycystic Ovary, *OA* Oligo/annovulation, *MD* Menstrual irregulation, *HA* Hypreandrogenism, *PCR-SSCP* Polymerase Chain Reaction-Single Strand Conformation Polymorphism, *PCR–RFLP* Polymerase Chain Reaction- Restriction Fragment Length Polymorphism, *PCR-SSP* Polymerase Chain Reaction- Sequence Specific Priming, *AES* Androgen Excess Society

**Table 2:** Representation of genotype frequencies of *FSHR* polymorphisms included studies

	**rs6165**						**HWE *p*- value**	**rs6166**						**HWE *p*- value**
	**Cases**			**Controls**				**Cases**			**Controls**			
	**Thr/Thr**	**Thr/Ala**	**Ala/Ala**	**Thr/Thr**	**Thr/Ala**	**Ala/Ala**		**Asn/Asn**	**Asn/Ser**	**Ser/Ser**	**Asn/Asn**	**Asn/Ser**	**Ser/Ser**	
**Conway et al. **[27]	**22**	**47**	**24**	**8**	**25**	**18**	**0.88**	**23**	**48**	**22**	**18**	**25**	**8**	**0.88**
**Tong et al. **[28]	**53**	**56**	**15**	**102**	**110**	**24**	**0.47**		**-**	**-**	**-**	**-**	**-**	**-**
**Sudo et al. **[29]	**3**	**12**	**3**	**73**	**73**	**22**	**0.57**	**3**	**12**	**3**	**73**	**73**	**22**	**0.57**
**Unsal et al. **[30]	**16**	**19**	**9**	**16**	**25**	**9**	**0.88**	**13**	**20**	**11**	**14**	**27**	**9**	**0.2**
**Valkenburg et al. **[17]	**-**	**-**	**-**	**-**	**-**	**-**	**-**	**123**	**248**	**124**	**782**	**1500**	**630**	**0.07**
**Du et al. **[31]	**26**	**20**	**9**	**40**	**37**	**15**	**0.2**	**26**	**26**	**3**	**40**	**34**	**16**	**0.07**
**Gu et al. **[16]	**81**	**116**	**38**	**50**	**56**	**22**	**0.35**	**138**	**91**	**6**	**92**	**35**	**1**	**0.23**
**Mohiyiddeen et al. **[32]	**-**	**-**	**-**	**-**	**-**	**-**	**-**	**14**	**34**	**10**	**20**	**47**	**16**	**0.21**
**Fu et al. **[33]	**192**	**156**	**36**	**362**	**329**	**77**	**0.86**	**187**	**162**	**35**	**357**	**334**	**77**	**0.93**
**Kambalachenu et al. **[34]	**-**	**-**	**-**	**-**	**-**	**-**	**-**	**25**	**64**	**8**	**31**	**52**	**18**	**0.63**
**Liaqat et al. **[35]	**27**	**47**	**22**	**22**	**49**	**25**	**0.83**	**29**	**47**	**20**	**24**	**47**	**25**	**0.83**
**Singhasena et al. **[36]	**70**	**53**	**10**	**70**	**56**	**6**	**0.20**	**69**	**59**	**5**	**72**	**54**	**6**	**0.29**
**Wu et al. **[37]	**93**	**95**	**27**	**91**	**100**	**14**	**0.052**	**93**	**94**	**28**	**94**	**98**	**13**	**0.057**
**Almawi et al. **[38]	**-**	**-**	**-**	**-**	**-**	**-**	**-**	**64**	**92**	**47**	**52**	**107**	**52**	**0.83**
**Thathapudi et al. **[39]	**-**	**-**	**-**	**-**	**-**	**-**	**-**	**74**	**99**	**31**	**44**	**90**	**70**	**0.14**
**Kim et al. **[4]	**145**	**176**	**56**	**181**	**176**	**31**	**0.18**	**149**	**178**	**50**	**180**	**176**	**32**	**0.22**
**Branavan et al. **[40]	**16**	**26**	**13**	**28**	**53**	**29**	**0.7**	**16**	**26**	**13**	**28**	**53**	**29**	**0.7**
**Wan et al. **[23]	**175**	**175**	**50**	**210**	**222**	**48**	**0.33**	**176**	**178**	**46**	**218**	**215**	**47**	**0.56**
**Kaur et al. **[24]	**93**	**175**	**153**	**76**	**146**	**100**	**0.11**	**119**	**198**	**104**	**92**	**156**	**74**	**0.6**
**Vieira et al. **[25]	**-**	**-**	**-**	**-**	**-**	**-**	**-**	**28**	**43**	**17**	**30**	**32**	**18**	**0.104**

*HWE* Hardy–Weinberg equilibrium

*p*-value < 0.05 considered as statistically significant

### Pooled analysis

**Fig. 2:**
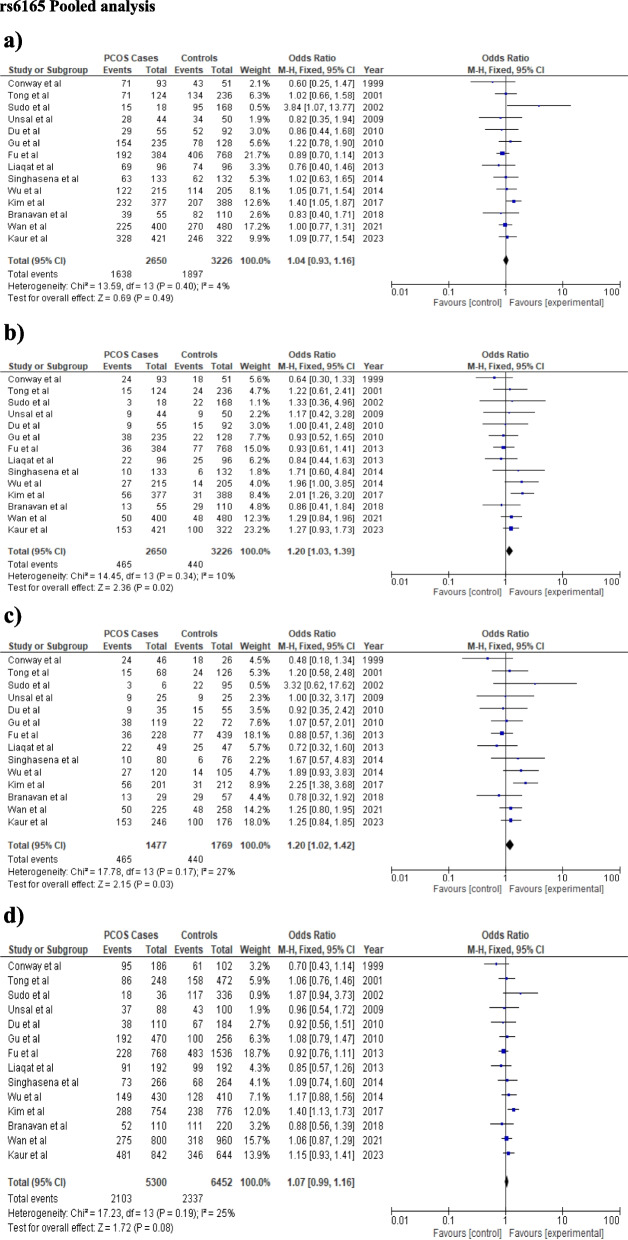
The association between *FSHR* (rs6165) variant and PCOS development using different genetic models in overall analysis: **a** Dominant model (GG + AG vs AA), **b** Recessive model (GG vs AG + AA), **c** Additive model (GG vs AA), **d** Allele model (G vs A). In each model, solid squares represent the OR and horizontal lines represent 95%CI and diamond represents the pooled OR and 95%CI

**Fig. 3:**
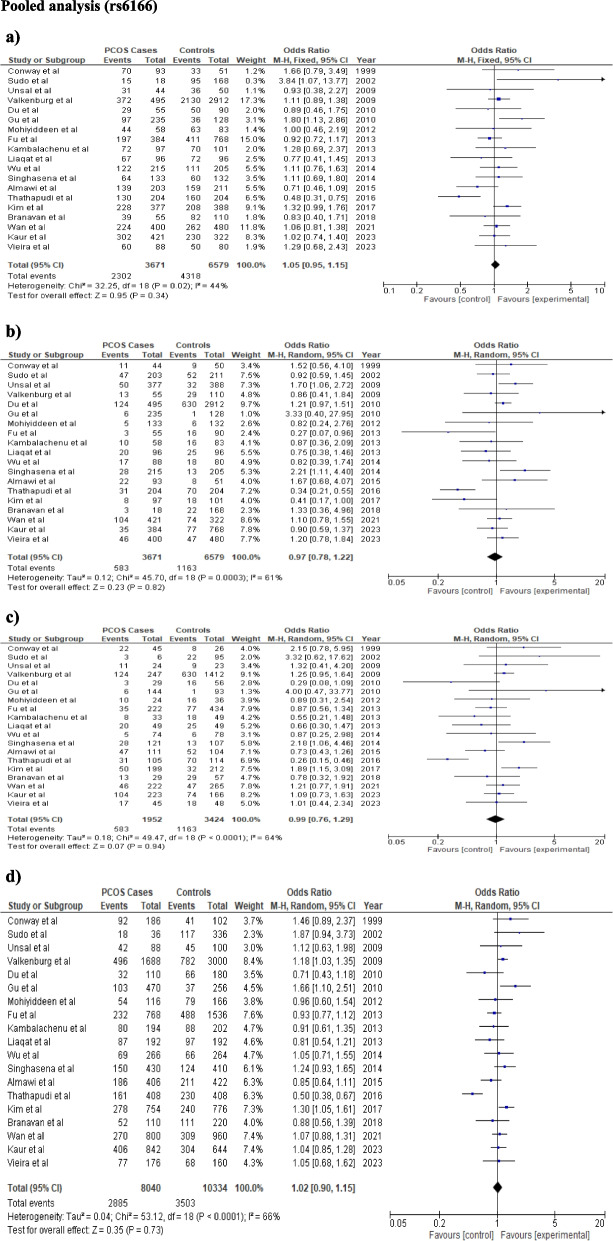
The association between *FSHR* (rs6166) variant and risk of PCOS using different genetic models in overall analysis: **a** Dominant model (GG + AG vs AA), **b** Recessive model (GG vs AG + AA), **c** Additive model (GG vs AA), **d** Allele model (G vs A). In each model, solid squares represent the OR and horizontal lines represent 95%CI and diamond represents the pooled OR and 95%CI

The findings of the meta-analysis were displayed in Table 3. For rs6165, fixed effect model was chosen for all the genetic models due to low heterogeneity. None of the genetic models confers a significant risk to PCOS in the overall analysis for rs6165 polymorphism (dominant model: OR = 1.04, CI: 0.93–1.16, *p* = 0.49; recessive model: OR-1.19 CI:1.03–1.3, *p* = 0.02; additive model: OR-1.2 CI:1.02–1.42, *p* = 0.03, allele model: OR = 1.07, CI: 0.99–1.18, *p* = 0.08 respectively) (Table 3; Fig. 2). Significant heterogeneity was observed for the rs6166 polymorphism in the allele, additive, and recessive models (I^2^ = 61%, 64%, 64%, respectively); however, genetic models did not indicate any risk for the development of PCOS (dominant model: OR = 1.05, CI:0.95–1.15, *p* = 0.34; Recessive model: OR = 0.97, CI:0.78–1.22, *p* = 0.82; Additive model: OR = 0.99, CI:0.76–1.29, *p* = 0.94; Allele model: OR = 1.02, CI: 0.90–1.15, *p* = 0.73) (Table 3, Fig. 3).

**Table 3:** Illustration of pooled and sub-group analysis under different genetic models

	**Overall Analysis**			**Asian Studies Meta-analysis**	**Indian Studies** **Meta-analysis**	**Other studies Meta-analysis**
	**FEM**	**REM**	**I**^**2**^			
**rs6165**	**OR (CI), *p*-value**					
Dominant Model	1.04(0.93–1.16), 0.49	1.19 (0.92–1.16), 0.54	4%	1.04(0.92–1.18), 0.53	1.09(0.77–1.54), 0.63	0.6(0.25–1.47), 0.26
Recessive Model	1.19(1.03–1.3), 0.02	1.2 (1.01–1.4), 0.04	10%	1.22(1.02–1.46), 0.03	1.27(0.93–1.73), 0.13	0.64(0.3–1.33), 0.23
Additive Model	1.2(1.02–1.42), 0.03	1.19(0.96–1.47), 0.11	27%	1.23(1.02–1.49), 0.03	1.25(0.84–1.85), 0.16	0.48(0.18–1.34), 0.16
Allele Model	1.07(0.99–1.18), 0.08	1.07(0.97–1.17), 0.19	25%	1.07(0.97–1.19), 0.18	1.15(0.93–1.41), 0.19	0.7(0.43–1.14), 0.16
**rs6166**						
Dominant Model	1.05(0.95–1.15), 0.34	1.04 (0.91–1.2) 0.56	44%	1.07 (0.95–1.2), 0.28	0.84 (0.66–1.06), 0.15	1.15 (0.95–1.39), 0.16
Recessive Model	1.02(0.91–1.15), 0.72	0.97 (0.78–1.22), 0.82	61%	1.11 (0.92–1.32), 0.27	0.7 (0.54–0.9), 0.006*	1.02 (0.91–1.15), 0.11
Additive Model	1.03 (0.90–1.2), 0.67	0.99 (0.76–1.29), 0.94	64%	1.1 (0.91–1.34), 0.33	0.65 (0.48–0.89), 0.006*	1.25 (0.98–1.59), 0.07
Allele Model	1.03(0.96–1.10), 0.4	1.02 (0.90–1.15), 0.78	64%	1.06 (0.95–1.15), 0.19	0.82 (0.7–0.95), 0.01*	1.17 (1.04–1.32), 0.01*

*OR* Odds Ratio, *CI* Confidence Interval, *FEM* Fixed Effect Model, *REM* Random Effect Model

^*^p-value was considered significant after Bonferroni correction,

### Subgroup analysis

**Fig. 4:**
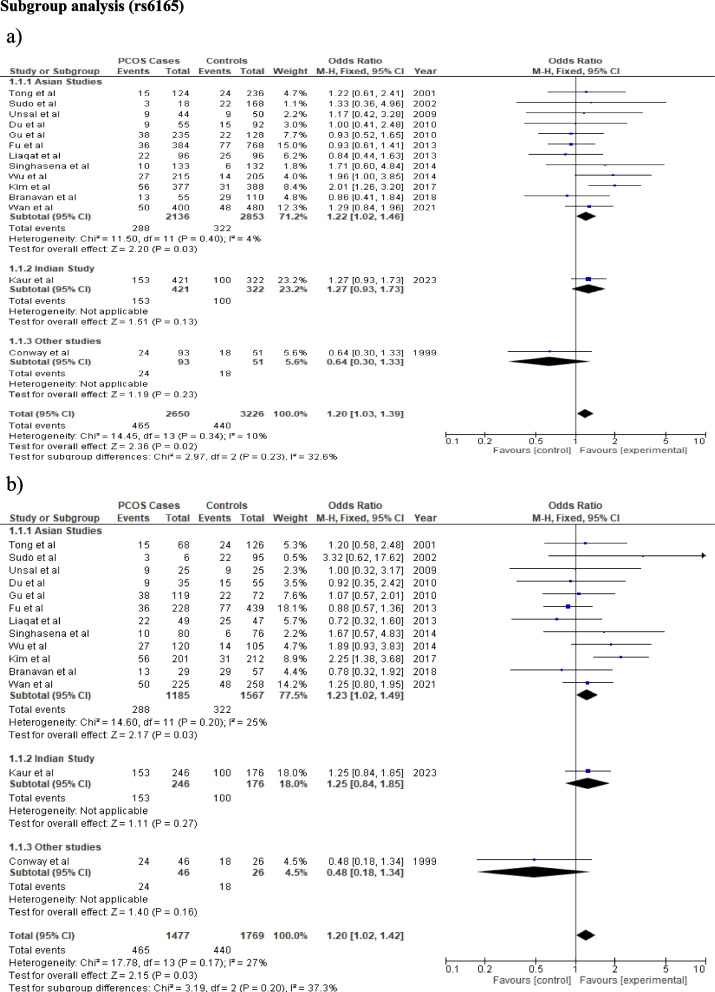
The association between *FSHR* (rs6165) and PCOS progression using different genetic models in Sub-group analysis: **a** Recessive model (GG vs AG + AA), **b** Additive model (GG vs AA). In each model, solid squares represent the OR and horizontal lines represent 95%CI and diamond represents the pooled OR and 95%CI

**Fig. 5:**
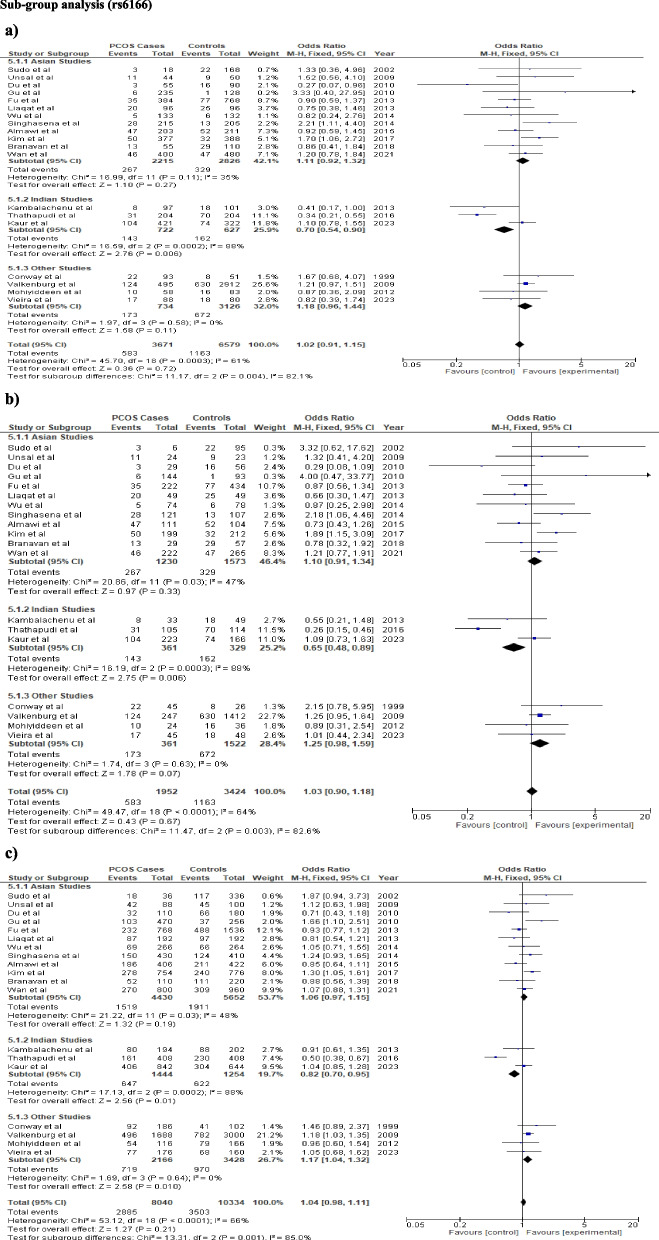
The association between *FSHR* (rs6166) variant and PCOS risk using different genetic models in overall analysis: **a** Recessive model (GG vs AG + AA), **b** Additive model (GG vs AA), **c** Allele model (G vs A). In each model, solid squares represent the OR and horizontal lines represent 95%CI and diamond represents the pooled OR and 95%CI

After the stratification by ethnicity, rs6165 polymorphism did not showed a significant risk of PCOS development in any ethnic group under any genetic model (Fig. 4; Table 3). In the Indian population, rs6166 polymorphism provides significant protection under recessive, additive, and allele models (Recessive model: OR = 0.7, CI:0.54–0.9, *p* = 0.006, Additive model: OR = 0.65, CI:0.48–0.89, *p* = 0.006, Allele model: OR = 0.82, CI:0.7–0.95, *p* = 0.01), while dominant model showed no association (Dominant model: OR = 0.84, CI:0.66–1.06, *p* = 0.15). Furthermore, a significant association was also found under the allelic model in other studies (Caucasian studies) (OR = 1.17, CI:1.04 -1.32, *p* = 0.01). However, none of the genetic models show any association with the Asian population (Table 3, Fig. 5).

### Publication bias and sensitivity of meta-analysis

Begg’s funnel plots were used to analyze the publication bias in included studies and, because of their symmetrical design, neither of the studies showed any signs of publication bias. Leave one out sensitivity analysis was performed to check the stability of the study. After systematically excluding each study, statistically comparable results were still obtained, showing statistically valid findings from our meta-analysis.

## Discussion

One of the most prevalent endocrine-metabolic disorders in women of reproductive age is PCOS and anovulation, infertility, and hyperandrogenism are its characteristic featu6res. PCOS is a polygenic condition, and numerous genetic variations are linked to its susceptibility [23]. The involvement of genetic factors in PCOS pathogenesis is evident in familial and genome-wide association studies (GWAS) [38, 41–43]. *FSHR*, *LHCGR*, *THADA*, and *DENND1A* are PCOS-susceptibility loci [41, 44].

Chronic anovulation is a hallmark of PCOS and the mechanism by which follicle selection is blocked in PCOS is still not known. An abnormal endocrine environment may be responsible for the premature arrest of some follicles and the advancement of follicle maturation in others. The suppression of FSH is the primary cause of this “suspension” of follicle growth and it had been reported that after careful administration of low-dose FSH, growth and ovulation of a healthy dominant follicle was re-established [45–48]. FSH performs by activating a specific receptor (FSHR) on the granulosa cells of the ovary. It was reported that phosphorylation of the Ser and Thr residues in the intracellular domain of FSHR may affect how the protein decouples from adenylyl cyclase. As a result, the function of the receptor, including the efficacy of FSH, may be affected by amino acid alterations linked to the corresponding SNPs (rs6165 and rs6166) [30, 49]. These two polymorphisms are located in exon 10 (rs6165 and rs6166) and are widely studied in different populations [4]; but the results had been inconsistent.

A meta-analysis is a statistical tool used to combine the findings of multiple studies on the same topic, increasing the statistical power to resolve discrepancies, and *FSHR* polymorphisms have already been meta-analyzed earlier as well [21, 22]. Additional studies on *FSHR* polymorphisms were found, after thoroughly reviewing the literature. Therefore, the present meta-analysis aims to investigate the association of *FSHR* exon 10 (rs6165 and rs6166) polymorphisms with PCOS risk. It comprises a total of 20 studies (Table 1). For rs6165, 14 studies were selected which include 2650 PCOS cases and 3226 controls and it was found that rs6165 does not exhibit an association with PCOS in any genetic model (Table 3). For rs6166, 19 studies with 3671 PCOS cases and 6579 controls were chosen and the protective role of Asn680Ser was observed in the Indian population under recessive, additive and allelic model while in the Caucasian population, it was demonstrated that Ser680 provides low to moderate risk under allelic model (OR-1.17, CI- 1.04–1.32, *p* = 0.01), however, in the Asian population, rs6166 polymorphism remained non-associated.

Chen et al. [21] conducted a meta-analysis on rs6165 and rs6166 that included 10 studies with 1720 PCOS cases and 4523 controls for rs6166 and 1097 cases and 1545 controls for rs6165. However, they did not observe any PCOS risk associated with *FSHR* polymorphisms. Another meta-analysis was carried out by Qiu et al. [22] with 11 studies on Thr307Ala (1326 cases and 3867 controls) and Asn680Ser (1344 cases and 3885 controls) and their study reported that Asn680Ser under homozygote model and Asn allele might have a protective effect against PCOS. In the sub-group analysis, the Asn680 allele showed a protective role only in Caucasians, not in Asian PCOS women. Wan et al. [23] also did a meta-analysis and they included 8 articles published on the Asian population. The results of a pooled meta-analysis in Asians supported that rs6166 polymorphism was strongly related to PCOS susceptibility. They also did a subsequent stratified study and observed that rs6165 remained unrelated to PCOS susceptibility in Chinese and Koreans, while rs6166 was related to PCOS susceptibility in Koreans but not in Chinese.

## Conclusion

Our meta-analysis is the most comprehensive on the *FSHR* polymorphisms and PCOS risk. We scored each article using the Newcastle–Ottawa Quality Assessment Scale in order to find higher-quality publications, and each study included in the current meta-analysis received a rating of at least five. We examined all the included studies for fixed or random effect models and analyzed the total effects under dominant, recessive, additive, and allele models. This meta-analysis includes a higher number of studies than the earlier ones, thus it provides accurate estimation. In the present meta-analysis, it was concluded that polymorphism rs6166 was found to have a modest impact on PCOS, however, on a specific cohort. In a meta-analysis, if the degree of heterogeneity rises, it gets harder to justify an integrated conclusion. Heterogeneity for rs6166 polymorphism is higher despite of subgroup analysis, therefore these results cannot be generalized. Further studies on homogeneous and larger populations with ethnicity-matched controls are required to strengthen the statistical power and to better understand the role of *FSHR* polymorphisms with PCOS.

## Data Availability

NA.

## Abbreviations

PCOS: Polycystic Ovary Syndrome
LH: Luteinizing Hormone
ESHRE: European Society of Human Reproduction and Embryology
ASRM: American Society of Reproductive Medicine
FSH: Follicle Stimulating Hormone
GnRH: Gonadotropic Releasing Hormone
FSHR: Follicle Stimulating Hormone Receptor
LHCGR: Luteinizing Hormone/Choriogonadotropin Receptor
PCOSKB: Polycystic Ovary Syndrome Knowledge Base
MeSH: Medical Subject Headings
HWE: Hardy Weinberg Equilibrium
SNP: Single Nucleotide Polymorphism
NOS: New-Castle Ottawa Scale
PRISMA: Preferred Reporting Items for Systematic Reviews and Meta-Analysis
REM: Random Effect Model
FEM: Fixed Effect Model
PCO: Polycystic Ovary
OA: Oligo/anovulation
MD: Menstrual irregulation
HA: Hyperandrogenism
PCR-SSCP: Polymerase Chain Reaction-Single Strand Conformation Polymorphism
PCR–RFLP: Polymerase Chain Reaction- Restriction Fragment Length Polymorphism
PCR-SSP: Polymerase Chain Reaction- Sequence Specific Priming
OR: Odds Ratio
CI: Confidence Interval
THADA: Thyroid Adenoma Associated
DENDD1A: DENN domain-containing protein 1A
